# Items

**Published:** 1846-02-01

**Authors:** 


*Items.*—We learn that the Medical Schools, generally have large classes the present winter. We shall endeavor to obtain authentic statistics for our next number.—The New York Medical Intelligencer has beeen discontinued for want of patronage.—In preparing the syrup of the Iodide of Iron, according to the formula given in our last number, caution is required. Mr. Mathews, Druggist of this city, was exposed to considerable danger from the bursting of the bottle in which the iron wire, iodine, and water were agitated. He was attempting, however, to prepare a much larger quantity than the formula specified. There is no danger in uniting the quantities of the ingredients mentioned in the article referred to, provided a strong bottle be selected.—A suit was recently brought in Poughkeepsie, N. Y., for a bill for medical services rendered by a Clairvoyant. The case was decided for the defendant, on the ground that the system involves deception and fraud. If this be a valid defence why does it not apply to other systems of empiricism which are equally deceptive and fraudulent, e. g. homœopathy? We have no desire, however, to see this plea successful; those who deliberately commit the folly of patronizing every new fangled humbug should be compelled to foot the bills.—Dr. Lawson, Prof. of Path. Anatomy in Transylvania University, and editor of the Western Lancet, has recently returned from a foreign tour, and resumed his editorial labors.—We regret to see by the Lancet, and an extra from a Lexington newspaper,that a want of harmony exists between the Transylvania and Louisville schools.—Prof. Mitchell states in an introductory address which has been published by the Journal, that numerous applications have been made for the vacant chair at Lexington. The appointment was to have been made on the 30th of January.—We have received the first number of a publication entitled the monthly Miscellany and Journal of Health; edited by W. M. Cornell, M. D., Boston, Mass. It is to be devoted to all subjects connected directly or remotely with the physical welfare of society. It is afforded at the low rate of $1 00. We wish the Editor success in his enterprise; but we are fearful that a popular periodical on the subject of health will not succeed without being spiced with Grahamism, Water cure, or some fashionable or practical humbug.

